# FS-15衰弱评分在骨髓增生异常综合征患者中的预后评估价值研究

**DOI:** 10.3760/cma.j.cn121090-20250303-00107

**Published:** 2025-09

**Authors:** 昕 王, 铁军 秦, 泽锋 徐, 士强 曲, 冰 李, 丽娟 潘, 清妍 高, 蒙 焦, 玥 仲, 彬涵 蒋, 琳琳 刘, 金影 赵, 文君 解, 志坚 肖

**Affiliations:** 1 中国医学科学院血液病医院（中国医学科学院血液学研究所），血液与健康全国重点实验室，国家血液系统疾病临床医学研究中心，细胞生态海河实验室，天津 300020 State Key Laboratory of Experimental Hematology, National Clinical Research Center for Blood Diseases, Haihe Laboratory of Cell Ecosystem, Institute of Hematology & Blood Diseases Hospital, Chinese Academy of Medical Sciences & Peking Union Medical College, Tianjin 300020, China; 2 天津医学健康研究院，天津 301600 Tianjin Institutes of Health Science, Tianjin 301600, China

**Keywords:** 骨髓增生异常综合征, 衰弱, 预后, IPSS-M, IPSS-R, Myelodysplastic syndrome, Frail, Prognosis, IPSS-M, IPSS-R

## Abstract

**目的:**

明确修订后的15项骨髓增生异常综合征衰弱评分（Revised 15-item MDS-specific frailty scale，FS-15）对中国骨髓增生异常综合征（MDS）患者的预后评估价值。

**方法:**

回顾性分析2016年8月至2023年6月就诊于中国医学科学院血液病医院的812例初治MDS患者，应用FS-15进行评分，根据结果将其分为衰弱及非衰弱两组，分析不同组别患者的临床和实验室特征及总生存（OS）情况。

**结果:**

①患者的中位年龄55（*IQR* 45～64）岁，中位随访时间22.5（95％ *CI*：20.2～24.9）个月，中位OS时间43.3（95％ *CI*：36.8～49.8）个月。患者FS-15得分中位数为0.42分，截断值为0.44分。男性患者得分相对女性较高（0.42分对0.38分，*P*＝0.006），修订版国际预后评分系统（IPSS-R）（*P*＝0.001）和分子国际预后评分系统（IPSS-M）（*P*＝0.014）分组中，极高风险组患者FS-15得分均显著高于极低风险组。②非衰弱组患者（452例）的中位OS时间为54.7（95％ *CI*：47.5～NA）个月，衰弱组（360例）为31.5（95％ *CI*：22.9～41.0）个月，3年OS率分别为（63.2±3.2）％和（46.4±3.6）％，5年OS率分别为（49.9±4.7）％和（32.0±4.3）％，差异有统计学意义（*P*<0.001）。③在IPSS-M低危及极高危患者中，非衰弱组患者的3年OS率明显高于衰弱组（均*P*<0.05）；在IPSS-R极低危、低危及高危患者中，非衰弱组患者的3年OS率明显高于衰弱组（均*P*<0.05）。④在接受去甲基化药物治疗的患者中，非衰弱组总体有效率显著高于衰弱组（86.7％对64.6％，*P*＝0.007）；治疗相关不良反应方面，衰弱组粒细胞缺乏伴发热（67.1％对47.4％，*P*＝0.016）及肝功能异常（30.0％对14.5％，*P*＝0.023）的发生率均高于非衰弱组。

**结论:**

应用FS-15对中国初治MDS患者进行衰弱评估具备可行性，并且可作为有效的预后评估工具使用。

## Abstract (en)

**Objective:**

To identify the prognostic value of the Revised 15-item Myelodysplastic Syndrome-specific frailty scale（FS-15）in Chinese patients with myelodysplastic syndromes（MDS）.

**Methods:**

This retrospective study analyzed 812 patients with newly diagnosed MDS admitted to the Institute of Hematology and Blood Diseases Hospital, Chinese Academy of Medical Sciences, and Peking Union Medical College from August 2016 to June 2023. Patients were assessed using the FS-15 and subsequently categorized into frail and non-frail groups. Clinical and laboratory characteristics, as well as overall survival（OS）, were compared between these groups.

**Results:**

① The median patient age was 55 years（*IQR* 45–64）, with a median follow-up of 22.5 months（95％ *CI*: 20.2–24.9）and a median OS of 43.3 months（95％ *CI*: 36.8–49.8）. The median FS-15 score was 0.42, with a cutoff value of 0.44. Male patients demonstrated higher median FS-15 scores than female patients（0.42 *vs* 0.38, *P*＝0.006）. In both the Revised International Prognostic Scoring System（IPSS-R; *P*＝0.001）and Molecular International Prognostic Scoring System（IPSS-M; *P*＝0.014）stratifications, FS-15 scores were significantly higher in the very high-risk group compared with the very low-risk group. ② The median OS was 54.7 months（95％ *CI*: 47.5–NA）and 31.5 months（95％ *CI*: 22.9–41.0）in the nonfrail（*n*＝452）and frail groups（*n*＝360), respectively（*P*<0.001）. The 3-year OS rates were（63.2±3.2）％ and（46.4±3.6）％ for the non-frail and frail groups, with 5-year OS rates of（49.9±4.7）％ and（32.0±4.3）％, respectively（*P*<0.001). ③Subgroup analysis revealed that nonfrail patients demonstrated significantly higher 3-year OS rates than frail patients in both the IPSS-M low-risk and very high-risk groups（all *P*<0.05）. Similarly, nonfrail patients demonstrated superior 3-year OS rates compared with frail patients in the IPSS-R very low-risk, low-risk, and high-risk groups（all *P*<0.05）. ④Among patients receiving hypomethylating agent therapy, the overall response rate was significantly higher in the non-frail group than in the frail group（86.7％ *vs* 64.6％, *P*＝0.007）. Moreover, the frail group experienced higher rates of treatment-related adverse events, including febrile neutropenia（67.1％ *vs* 47.4％, *P*＝0.016）and liver function abnormalities（30.0％ *vs* 14.5％, *P*＝0.023）, compared with the non-frail group.

**Conclusion:**

The FS-15 frailty score is a feasible and effective tool for assessing frailty in patients newly diagnosed with MDS in China and serves as a valuable prognostic indicator.

骨髓增生异常综合征（Myelodysplastic syndrome, MDS），亦称骨髓增殖性肿瘤（Myelodysplastic neoplasms, MDS），是一组主要发生于老年人的髓系肿瘤。MDS具有高度异质性，精准的预后评估和危险分层对于指导临床决策至关重要。目前，MDS的主要预后评分系统是基于血细胞减少程度、骨髓原始细胞比例、细胞遗传学和基因突变等疾病相关因素提出的修订版国际预后评分系统（Revised International Prognostic Scoring System, IPSS-R）[1]和分子国际预后评分系统（the Molecular International Prognostic Scoring System, IPSS-M）[2]–[3]。患者相关的因素，如合并疾病指数和衰弱（Frailty）程度等对疾病的预后影响越来越受到重视。此前我们已证实合并疾病是独立于IPSS-R的预后影响因素[4]，最近我们采用修订后的15项MDS特异性衰弱评分量表（FS-15）[5]对初治MDS患者进行了评估，探讨FS-15衰弱评分在MDS患者中的预后价值。

## 病例与方法

1. 病例资料：本研究为回顾性队列研究，对2016年8月至2023年6月就诊于中国医学科学院血液病医院MDS和MPN诊疗中心的初治MDS患者进行分析，共812例患者可采集到FS-15条目。所有患者诊断均符合WHO 2016标准[6]。基于IPSS-R和IPSS-M对患者进行危险分层。

2. FS-15衰弱评分：FS-15衰弱评分为包括15项指标的衰弱指数（Frailty Index, FI），每项指标按照预设标准进行赋值（表1），15项指标中缺失数小于20％的患者可进行评分，通过网址https://qxcalc.app.link/mdsfrailty在线计算FS-15得分[5]。由于本研究为回顾性研究，812例患者均缺失疲劳评分和4米步行试验的条目。得分计算方法为：存在缺陷的条目的累积评分/患者所具有的缺陷条目的总数。FS-15得分范围为0～1，数值越高代表个体存在的缺陷数越多，即衰弱程度越高。本研究与FS-15研究中加拿大队列（Myelodysplastic syndromes-Canada, MDS-CAN）的患者各条目缺陷率如表1所示。绘制FS-15得分预测患者总生存（OS）的受试者操作特征曲线（ROC）并计算曲线下面积（AUC）。根据约登指数确定FS-15得分的截断值为0.44分，将患者分为衰弱（FS-15>0.44分）及非衰弱（FS-15 ≤ 0.44分）组。

**表1:** FS-15衰弱评分标准及患者各条目缺陷率（％）

项目	缺陷定义	权重	本中心队列缺陷率（812例）	MDS-CAN队列缺陷率（433例）
红细胞分布宽度（RDW）	若超出医院实验室正常范围，则判定为缺陷	2	86.3	77.8
乳酸脱氢酶（LDH）	若超出医院实验室正常范围，则判定为缺陷	2	37.3	34.4
白细胞计数（WBC）	若超出医院实验室正常范围，则判定为缺陷	1	82.7	63.4
平均红细胞体积（MCV）	若超出医院实验室正常范围，则判定为缺陷	1	53.0	56.0
铁蛋白（Ferritin）	若超出医院实验室正常范围，则判定为缺陷	1	64.0	52.4
网织红细胞计数（Rct）	若超出医院实验室正常范围，则判定为缺陷	1	43.9	30.3
碱性磷酸酶（ALP）	若超出医院实验室正常范围，则判定为缺陷	1	14.7	10.5
丙氨酸转氨酶（ALT）	若超出医院实验室正常范围，则判定为缺陷	1	4.3	6.1
总胆红素（TBIL）	若超出正常医院实验室上限（ULN）的1.5倍，则判定为缺陷	1	4.9	1.9
肌酐清除率	<60 ml/min判定为缺陷	1（<30 ml/min）； 0.5（30～59 ml/min）	4.4	19.2
身体质量指数（BMI）	<18.5或≥25.0判定为缺陷	1（<18.5或≥30.0）； 0.5（25.0～<30.0）	49.4	47.8
既往或当前肿瘤病史	若当前或既往患有恶性肿瘤，则判定为缺陷	1	3.0	20.1
独立规划、准备及提供适当餐食的能力	若不能独立完成，则判定为缺陷	1	3.0	5.5
疲劳评分	≥4分判定为缺陷	1（≥7分）； 0.5（4～6分）	缺失	38.2
4米步行试验（步行4米所需时间）	≥4 s判定为缺陷	1（>6.67 s）； 0.5（4～6.67 s）	缺失	32.1

3. 疗效评估及不良事件判定：对接受去甲基化药物（HMA）治疗的患者进行疗效评估，最佳疗效评估参照2006年国际工作组（IWG）修订的疗效评价标准[7]，分为完全缓解（CR）、骨髓完全缓解（mCR）、部分缓解（PR）、疾病稳定（SD）、血液学改善（HI）、治疗失败及复发。总体有效率（ORR）参照既往研究定义为CR率+mCR率+PR率+HI率[8]。根据CTCAE 5.0标准判定患者在接受治疗期间出现的血液学和非血液学不良反应，并记录因这些不良反应导致患者治疗中断、推迟或调整治疗计划的情况。

4. 随访：采用电话联系、查阅住院或门诊病历的方式对患者进行随访，末次随访时间为2023年9月1日。OS期定义为从确诊MDS到患者死亡或末次随访之日。

5. 统计学处理：采用R 4.4.2进行统计学分析和绘图。计量资料符合偏态分布，以中位数（四分位距）［*M*（*IQR*）］进行描述，组间比较使用Wilcoxon检验。计数资料间率的比较采用卡方检验或Fisher确切概率法。使用Kaplan-Meier法进行生存分析并绘制生存曲线，Log-rank检验比较组间差异。*P*<0.05为差异具有统计学意义。

## 结果

一、临床特征

共纳入812例患者为研究对象，男529例（65.1％），女283例（34.9％），中位年龄55（*IQR* 45～64）岁。依据IPSS-R将患者分为极低危（29例，3.5％）、低危（161例，19.8％）、中危（228例，28.0％）、高危（174例，21.4％）和极高危组（141例，17.3％）。依据IPSS-M将患者分为极低危（20例，2.5％）、低危（122例，15.0％）、较低危（107例，13.2％）、较高危（99例，12.2％）、高危（185例，22.8％）和极高危组（200例，24.6％）。另有79例（9.7％）患者缺失用于评估IPSS-R及IPSS-M的数据。

812例MDS患者中位FS-15得分为0.42（0.35，0.54）分。男性患者的FS-15得分高于女性患者［0.42（0.35，0.54）分对0.38（0.31，0.50）分，*P*＝0.006］；>60岁患者的FS-15得分高于≤60岁患者，但差异无统计学意义（*P*＝0.241）；IPSS-R（*P*＝0.001）和IPSS-M（*P*＝0.014）不同组别中的FS-15得分有差异，总体上较高风险组的得分相对较高（表2）。依据FS-15得分进行分组，其中衰弱组360例（44.3％），非衰弱组452例（55.7％）。衰弱组与非衰弱组患者相比，初诊时PLT及ANC差异无统计学意义，但HGB水平显著降低（*P*＝0.005）（表3）。

**表2:** FS-15得分在骨髓增生异常综合征患者不同性别、年龄、IPSS-R和IPSS-M组别中的分布（分）

临床特征	例数	平均值	中位数	*Q*1	*Q*3	最小值	最大值	*P*值
性别								0.006
男性	529	0.43	0.42	0.35	0.54	0.08	0.81	
女性	283	0.41	0.38	0.31	0.50	0.08	0.77	
年龄								0.241
> 60岁	285	0.43	0.42	0.35	0.54	0.08	0.81	
≤ 60岁	527	0.42	0.41	0.31	0.50	0.12	0.69	
IPSS-R分组^a^								0.001
极低危	29	0.35	0.35	0.25	0.46	0.15	0.53	
低危	161	0.42	0.42	0.31	0.54	0.12	0.77	
中危	228	0.42	0.42	0.35	0.54	0.08	0.81	
高危	174	0.44	0.42	0.35	0.54	0.19	0.81	
极高危	141	0.45	0.46	0.35	0.54	0.12	0.77	
IPSS-M分组^a^								0.014
极低危	20	0.33	0.29	0.19	0.43	0.12	0.77	
低危	122	0.41	0.42	0.31	0.50	0.15	0.77	
较低危	107	0.41	0.42	0.31	0.54	0.12	0.69	
较高危	99	0.44	0.42	0.35	0.54	0.15	0.81	
高危	185	0.43	0.42	0.35	0.54	0.08	0.73	
极高危	200	0.45	0.46	0.35	0.54	0.12	0.81	

总体	812	0.43	0.42	0.35	0.54	0.08	0.81	

**注** IPSS-R：修订版国际预后评分系统；IPSS-M：分子国际预后评分系统；*Q*1：第一四分位数；*Q*3：第三四分位数。^a^79例患者缺失用于评估IPSS-R及IPSS-M的数据

**表3:** FS-15评分衰弱组与非衰弱组患者基线临床特征比较

临床特征	所有患者（812例）	衰弱患者（360例）	非衰弱患者（452例）	*P*值
年龄［岁，*M*（*IQR*）］	56（45, 64）	55（46, 63）	56（45, 64）	0.755
性别［例（％）］				0.297
男性	529（65.1）	242（67.2）	287（63.5）	
女性	283（34.9）	118（32.8）	165（36.5）	
骨髓原始细胞比例［％, *M*（*IQR*）］	2.25（0, 6.5）	2.0（0, 5）	2.25（0, 7.5）	0.232
外周血细胞计数				
WBC［×10^9^/L, *M*（*IQR*）］	2.50（1.72, 3.59）	2.64（1.72, 3.62）	2.41（1.74, 3.57）	0.492
ANC［×10^9^/L, *M*（*IQR*）］	1.06（0.62, 1.89）	1.10（0.58, 1.91）	1.04（0.64, 1.85）	0.892
HGB［g/L, *M*（*IQR*）］	80（66, 96）	79（65, 91）	81（66, 101）	0.005
PLT［×10^9^/L, *M*（*IQR*）］	62（32, 123）	59（29, 124）	64（34, 120）	0.161
IPSS-R分组［例（％）］				0.084
极低危	29（3.5）	8（2.2）	21（4.6）	
低危	161（19.8）	68（18.8）	93（20.5）	
中危	228（28.0）	95（26.3）	133（29.4）	
高危	174（21.4）	80（22.2）	94（20.7）	
极高危	141（17.3）	74（20.5）	67（14.8）	
缺失	79（9.7）	35（9.7）	44（9.7）	
IPSS-M分组［例（％）］				0.151
极低危	20（2.5）	5（1.4）	15（4.0）	
低危	122（15.0）	50（13.9）	72（15.9）	
较低危	107（13.2）	40（11.1）	67（14.8）	
较高危	99（12.2）	44（12.2）	55（12.2）	
高危	185（22.8）	85（23.6）	100（22.1）	
极高危	200（24.6）	101（28.1）	99（21.9）	
缺失	79（9.7）	35（9.7）	44（9.7）	
WHO 2016诊断分型［例（％）］				0.529
MDS-SLD/MLD	327（40.3）	148（41.1）	179（39.6）	
MDS-RS-SLD/MLD	71（8.8）	30（8.3）	41（9.1）	
MDS-EB1/2	376（46.3）	169（47.0）	207（45.8）	
5q−综合征	10（1.2）	1（0.3）	9（2.0）	
MDS不能分类	28（3.4）	12（3.3）	16（3.5）	

**注** IPSS：修订版国际预后评分系统；IPSS-M：分子国际预后评分系统；MDS-SLD/MLD：MDS伴单/多系病态造血；RS：环状铁粒幼细胞；MDS-EB：MDS伴原始细胞增多

598例患者有治疗数据记录，其中208例（34.8％）接受了HMA治疗作为一线方案（地西他滨或阿扎胞苷，联合/不联合维奈克拉），45例（7.5％）患者接受了包括注射红细胞生成素、输注血细胞在内的支持治疗，141例（23.6％）患者接受免疫抑制治疗（环孢素A、沙利度胺及达那唑）。共有99例（16.6％）患者接受了异基因造血干细胞移植。中位随访22.5（95％ *CI*：20.2～24.9）个月，中位OS时间为43.3（95％ *CI*：36.8～49.8）个月。

二、FS-15不同分组患者生存情况

非衰弱组患者的中位OS时间为54.7（95％ *CI*：47.5～NA）个月，衰弱组为31.5（95％ *CI*：22.9～41.0）个月，3年OS率分别为（63.2±3.2）％和（46.4±3.6）％，5年OS率分别为（49.9±4.7）％和（32.0±4.3）％，差异有统计学意义（*P*<0.001）（图1）。

**图1:**
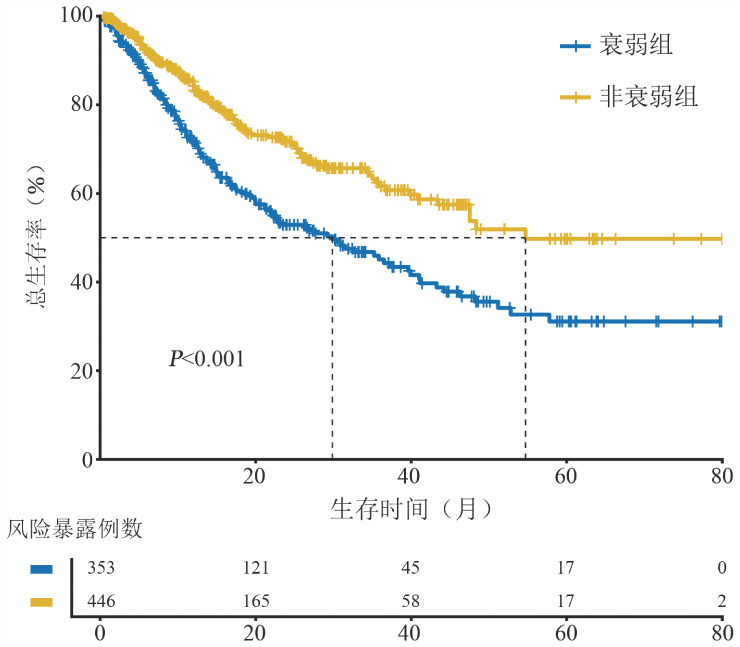
FS-15评分衰弱与非衰弱患者的总生存曲线比较

在IPSS-R不同危险组中对衰弱/非衰弱患者进行亚组生存分析发现（图2）：极低危组中，非衰弱患者的3年OS率显著高于衰弱患者［100.0％对（66.7±19.2）％，*P*＝0.008］；低危组中，非衰弱组患者的3年OS率同样显著高于衰弱组患者［（80.8±5.4）％对（56.7±7.9）％，*P*＝0.012］；高危组中，非衰弱组患者的3年OS率为（48.8±8.1）％，衰弱组为（26.8±6.8）％（*P*＝0.016）。但IPSS-R中危组和极高危组中，衰弱与非衰弱组的OS率差异无统计学意义。

**图2:**
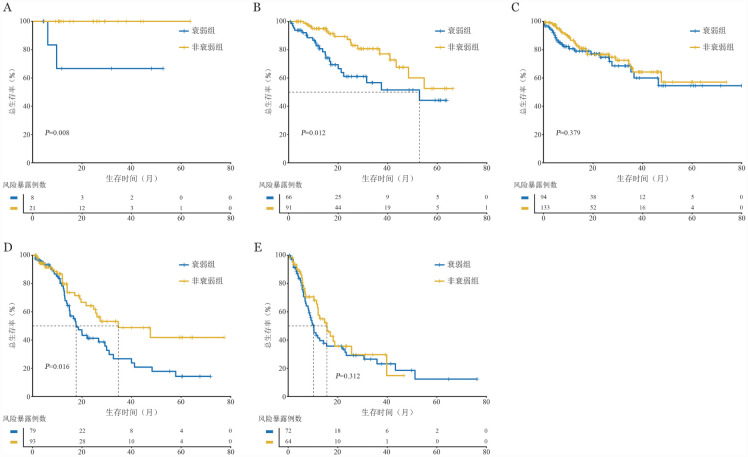
修订版国际预后评分系统（IPSS-R）不同组别中FS-15评分衰弱与非衰弱患者总生存曲线比较 **A** IPSS-R极低危组；**B** IPSS-R低危组；**C** IPSS-R中危组；**D** IPSS-R高危组；**E** IPSS-R极高危组

在IPSS-M组中亚组分析发现（图3）：低危患者中，非衰弱患者的3年OS率高于衰弱患者［（88.7±4.9）％对（66.3±7.8）％，*P*＝0.014］。在极高危组中，非衰弱患者的3年OS率也高于衰弱患者［（40.9±6.9）％对（25.4±5.4）％，*P*＝0.024］。其余组别患者中，非衰弱患者生存预后相较于衰弱组患者均较好，尤其是在极低危组患者中，非衰弱患者预后优于衰弱组患者，但可能由于样本量不足等原因，差异无统计学意义（*P*＝0.073）。

**图3:**
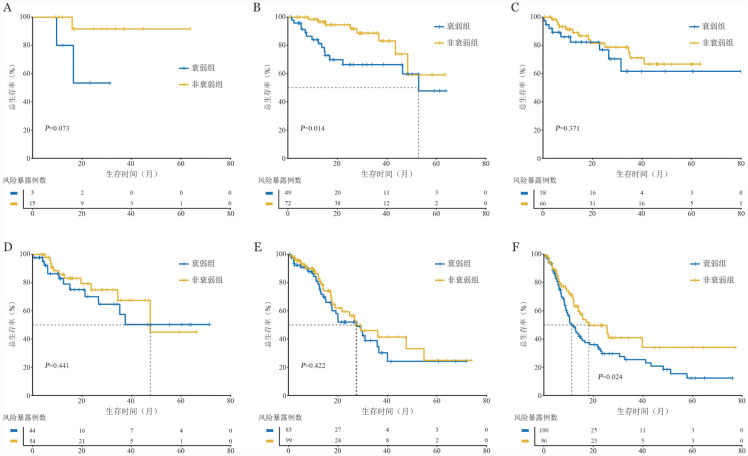
分子国际预后评分系统（IPSS-M）不同组别中FS-15评分衰弱与非衰弱患者总生存曲线比较 **A** IPSS-M极低危组；**B** IPSS-M低危组；**C** IPSS-M较低危组；**D** IPSS-M较高危组；**E** IPSS-M高危组；**F** IPSS-M极高危组

在骨髓原始细胞低于5％的患者中，衰弱患者的3年OS率明显低于非衰弱患者［（55.9±4.4）％对（74.9±3.7）％，*P*<0.001］（图4A），而在原始细胞较高（5％～9％及10％～19％）的患者中，尽管趋势相似，但差异无统计学意义（图4B、C）。

**图4:**
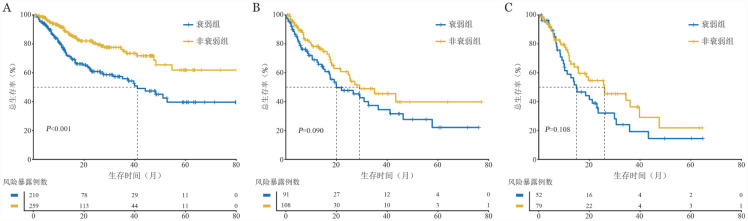
不同原始细胞比例组FS-15评分衰弱与非衰弱患者总生存曲线比较 **A** 原始细胞<5％组；**B** 原始细胞5％～9％组；**C** 原始细胞10％～19％组

对接受HMA治疗及接受免疫抑制剂联合促造血治疗的患者进行亚组分析，对比发现两组中非衰弱患者的3年OS率均高于衰弱患者［HMA治疗组：（43.2±7.4）％对（21.1±6.3）％，*P*＝0.008；免疫抑制剂联合促造血治疗组：（80.0±4.6）％对（66.7±5.9）％，*P*＝0.016］（图5）。

**图5:**
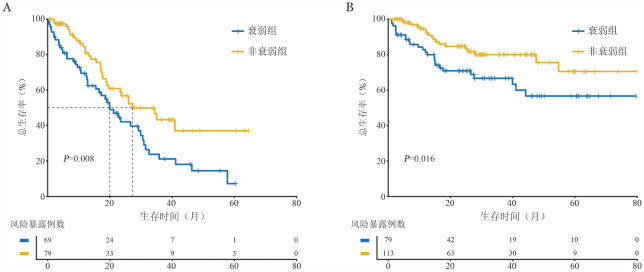
接受不同治疗组FS-15评分衰弱与非衰弱患者总生存曲线比较 **A** 去甲基化药物治疗组；**B** 免疫抑制剂联合促造血治疗组

三、FS-15不同分组患者HMA疗效及安全性比较

共有208例患者接受了HMA治疗，其中108例具有疗效评估数据，针对这部分患者进行了疗效分析，结果显示，衰弱组ORR为64.6％，非衰弱组ORR为86.7％，差异具有统计学意义（*χ*^2^＝7.310，*P*＝0.007）。

接受HMA治疗并记录有治疗相关不良反应的148例患者中，衰弱组67.1％（47/70）患者出现粒细胞缺乏（粒缺）伴发热，高于非衰弱组的47.4％（36/76）（*χ*^2^＝5.808，*P*＝0.016）。治疗后出现丙氨酸转氨酶、天冬氨酸转氨酶同时或单独升高的患者比例，衰弱组高于非衰弱组［30.0％（21/70），14.5％（11/76），*χ*^2^=5.133，*P*＝0.023］。

## 讨论

衰弱指机体在应对外界应激时功能储备下降，导致对不良事件的易感性增加[9]。恶性血液病患者的疾病相关临床表现，如血细胞减少、治疗相关的免疫抑制、感染等可能会加重患者的衰弱状态，同时衰弱又会进一步影响患者的预后、治疗耐受性及生活质量[10]–[13]。衰弱与血液恶性肿瘤之间存在着双向的影响关系。因此在恶性血液病患者中进行衰弱评估有助于全面了解患者的健康状况，指导个体化治疗方案的制定。目前临床常用的衰弱评估工具包括基于表型模型的Fried衰弱标准[9]以及基于缺陷累积模型的Rockwood衰弱指数[14]，在实体瘤患者中应用较为广泛，但在恶性血液病患者尤其是MDS患者中目前尚无统一的标准对患者进行衰弱评估，导致临床实践中衰弱筛查和管理的实施存在较大异质性。

MDS FS-15衰弱评分基于衰弱指数（FI），包括了血常规、生化、自理能力、疲劳指数及BMI等维度的指标，在MDS-CAN前瞻队列中进行开发及验证。本研究中患者的大部分指标缺陷比例略高于MDS-CAN队列，本队列中患者仅有13项指标进行评估的因素，衰弱得分的中位数仍相对较高，提示相较于MDS-CAN队列，本中心患者的整体衰弱程度可能更加严重。这一现象可能与患者的诊疗模式、社会经济背景及疾病特征等因素相关。我们近期的研究发现中国MDS患者的三系血细胞计数相较于国际预后工作组MDS（IWG-PM）2 191例患者普遍更低[15]，而较低的血细胞计数有可能导致MDS患者的衰弱程度加深[16]。此外，我国医疗体系中对衰弱管理的关注度相对不足，可能导致部分患者未能及时进行针对性干预。本研究显示，男性MDS患者的FS-15衰弱得分的中位数高于女性患者（*P*＝0.006），这一结果不同于普通老年人群研究中女性衰弱程度更高的普遍结论。既往研究提示由于雌激素水平下降等原因，女性的衰弱率更高，但男性的衰弱与死亡率之间的关联更强[17]。也有研究发现在特定人群中男性的衰弱程度可能更重[18]。MDS患者衰弱程度的性别差异机制仍需进一步研究。此外，> 60岁患者的衰弱得分高于≤ 60岁患者，但差异无统计学意义（*P*＝0.241），提示年龄虽然是衰弱发生的重要因素，但在本研究人群中，衰弱程度可能受到其他因素（如疾病风险分层、合并症）的更大影响。

本研究显示，IPSS-R和IPSS-M极高危患者的FS-15评分均显著高于极低危患者，提示在MDS患者中，疾病风险更高的患者，相较于低风险患者其衰弱程度可能更严重。这一趋势符合MDS疾病进展的规律，高危及极高危患者往往伴有更严重的骨髓衰竭、贫血及慢性炎症状态[19]，这些因素可能共同促进衰弱的发生和进展。

在衰弱与血液学指标的关系分析中，本研究发现衰弱组患者的HGB水平显著低于非衰弱组（*P*＝0.005），而PLT及ANC无明显差异。这一结果提示贫血可能是MDS患者衰弱发生的重要影响因素。贫血不仅影响氧输送，还可能导致疲劳、心功能下降及生活质量下降，进而加剧衰弱表现[20]。相比之下，血小板和中性粒细胞的减少可能更多影响出血及感染风险，而对衰弱的直接影响相对较小。因此，在MDS患者的衰弱管理中，贫血的早期干预可能具有重要的临床意义。但需注意的是，FS-15评分中已包含多个红系相关指标（如红细胞分布宽度、平均红细胞体积及网织红细胞计数），这些变量与HGB具有相关性，可能导致分析中出现共线性偏倚，从而夸大贫血与衰弱评分之间的关联程度。因此，该结论仍需在采用其他独立衰弱评估工具的研究中进一步验证。

整体来看非衰弱患者具有显著更长的OS期，并且在IPSS-R及IPSS-M不同风险分层中表现出一定的异质性。已有多项国外研究发现衰弱与MDS患者的低生存期密切相关，一项回顾性研究（114例）发现低血清白蛋白和较差的身体功能能够为IPSS提供额外的预后信息[21]。另一项前瞻性研究（445例）则发现衰弱可独立预测MDS患者的生存，且比合并症更具预测价值[22]。此外，一项欧洲多中心研究（195例）也得出日常活动受限和较差的生活质量/疲劳与MDS患者的预后不良相关的结论[23]。本研究进一步印证了这一观点。

在IPSS-R和IPSS-M极低危组和低危组中，非衰弱组的生存预后优于衰弱组，并且差异具有统计学意义。这表明即使是在整体预后相对较好的相对低危组中，衰弱状态仍能进一步区分患者的预后。可能是因为在疾病早期阶段，除了肿瘤负担外，衰弱作为反映全身健康状况的指标，其影响更易显现。相反，在中高危以及极高危患者组中，尽管衰弱的患者预后相对较差，但其影响可能被疾病本身的生物学特征所掩盖，因此差异不够显著。这一点在骨髓原始细胞不同比例患者的亚组生存分析中也得到了验证，可以发现在骨髓原始细胞比例较低的患者中，非衰弱患者预后显著优于衰弱患者，而在原始细胞比例较高的患者中，差异未达到统计学意义。同时我们认为部分组别患者例数较少也可能是造成差异无统计学意义的原因之一。

在治疗方案的亚组分析中，我们分别对接受HMA治疗和接受免疫抑制剂联合促造血治疗的患者进行了分析。结果显示无论采用哪种治疗方式，非衰弱患者的3年OS率均显著高于衰弱患者。在接受HMA治疗的患者中，衰弱组的ORR及OS率明显低于非衰弱组，并且衰弱组在治疗过程中出现粒缺伴发热及肝功能异常的比例均显著高于非衰弱组。这提示患者的衰弱，也就是机体功能储备不足可能会降低患者对于治疗的耐受性和疗效。在其他血液恶性肿瘤中也有类似的结果[24]。

因此，在制定MDS患者治疗方案时，除了传统的IPSS-R或IPSS-M风险分层外，应综合评估患者的衰弱状态，加入衰弱评估可以更精细地识别出预后较差的患者并制定个体化治疗方案。对于非衰弱的低危组患者，可考虑适度强度的治疗策略（如适量调整HMA的剂量或积极评估造血干细胞移植的可能性），以期获得更好的生存获益；而衰弱患者对于治疗相关毒性更为敏感，机体耐受性较低，则应更加注重支持性治疗和生活质量维护，采取相对保守的治疗方案，避免过度治疗带来的风险。

目前应用于血液肿瘤患者的衰弱评估工具包括衰弱老年人调查问卷（VES-13）[25]、G-8老年筛查工具[26]、临床衰弱量表（CFS）[27]等，尚无明确的针对MDS患者的统一衰弱评估方法。FS-15衰弱评分仅包含15项指标，获取数据较为方便，在临床实践中具备很高的实用性。通过在本中心812例患者中的验证，我们认为FS-15衰弱评分对中国MDS患者的危险分层有很好的区分及预后判断指导价值，可以为IPSS-R及IPSS-M预后评分提供有效的补充。本研究的不足之处有：为单中心回顾性研究，且有一定比例患者的条目缺失。今后应开展多中心、前瞻性研究，进一步验证FS-15衰弱评分在MDS预后评估中的独立作用，并探索通过针对性干预（如营养支持、运动康复等）改善衰弱状态、提高治疗耐受性和改善预后的可能性，从而推动MDS个体化治疗策略的进一步发展。
